# Assessment of Self-Medication Practices Among Medical, Pharmacy, and Health Science Students in Gondar University, Ethiopia

**DOI:** 10.4103/0975-1483.66798

**Published:** 2010

**Authors:** S M Abay, W Amelo

**Affiliations:** *Department of Pharmacy, Jimma University, Jimma, Ethiopia*; 1 *Department of Pharmacology, Faculty of Medicine, Addis Ababa University, Ethiopia*

**Keywords:** Drug, Ethiopia, self-medication, students

## Abstract

The study was aimed at assessing the magnitude and factors of self-medication among medical, pharmacy, and health science students of GCMHS (Gondar College of Medicine and Health Sciences). A cross-sectional study with two-month illness recall was conducted. A Questionnaire consisting of demographic questions and questions on illnesses in the last two months prior to the interview and treatment strategies was prepared and administered to the 414 students, selected as the sample population, from the GCMHS students. Of a total of 414 students, 213 (51.5%) reported at least one episode of an illness, and 82 (38.5%) of them practiced self-medication. Most drugs for self-medication were obtained from the pharmacy or drug shops; and the most commonly used drugs were Paracetamol and NSAIDs (Non-steroidal anti-inflammatory drugs). Common reported illnesses were fever and headache (24.8%) followed by cough and common cold (23.9%). Prior experience and the non-seriousness of the illness were the top two reported factors for self-medication. Reading materials were the top reported source of information. In conclusion, self-medication was practiced with a range of drugs from the conventional anti-pains to antibiotics. Although the practice of self-medication is inevitable; drug authorities and health professionals need to educate students about the pros and cons of self-medication.

## INTRODUCTION

In economically deprived countries most episodes of illness are treated by self-medication.[1] In a number of developing countries many drugs are dispensed over the counter without medical supervision. In this case, self-medication provides a lower cost-alternative for people who cannot afford the cost of clinical service.[2]

Studies revealed that the increase in self-medication was due to a number of factors. These included socioeconomic factors, lifestyle, ready access to drugs, the increased potential to manage certain illnesses through self-care, and greater availability of medicinal products.[3]

A study conducted in the southern part of Ethiopia showed that 15% of the persons with perceived illnesses practiced self-medication.[4] In another study conducted in Addis Ababa and central Ethiopia, the magnitude of self-medication was as high as 50%.[5] The study carried out in North West Ethiopia showed 27.2% self-medication prevalence in the study areas (i.e., Gondar, Dabark, and Kola-Diba).[6] The previous study, included households in Gondar, did not consider Medical, Pharmacy, and Health Science students, who differed from the general population of Gondar. They were exposed to knowledge about diseases and drugs, so they might be expected to behave differently. To our knowledge, there is no published data with regard to self-medication practice and the factors that affect the practice in GCMHS students. The objective of our study was, therefore, to assess the self-medication practice, assess common types of illnesses, and identify frequently used drugs and determinants of self-medication.

## METHODOLOGY

**Study site:** Study was carried out in GCMS, North West Ethiopia. It is the oldest health professional training institute in Ethiopia. It was established in 1954, as a Public Health College and training center. The college is a pioneer in training Health Officers, Community Nurses, Medical Laboratory Technicians, and Sanitarians. Since then, the college is expanding its scope of activities and currently it is training students in Human Medicine, Pharmacy, Nursing, Midwifery, Environmental Health, Physiotherapy, Medical Laboratory Technology, Optometry, Anesthesia and Occupational Health and Safety. The students get healthcare services mainly from the Gondar University Hospital and also from different private and governmental healthcare settings providing this service.[7]

**Study population:** TThe cross-sectional study was conducted on 414 students taken as a sample from 2485 students in GCMHS. The stratified sampling method was used to choose the respective number of students from each department and each study year during data collection. The sample size was determined according to the following assumption. As there was no previous study conducted in the study area of the college Medical and Health Science a 50% expected prevalence of self-medication and 2% of sample population was added to compensate for the loss.

**Data collection and analysis:** The pre-tested, semi-structured questionnaire was prepared. Data was collected from April 20 to May 15, 2007. The study subjects were informed that the information collected would be anonymous; and participation would be totally voluntary. The age, sex, and year of study were noted. The information regarding the type of medication, illness for which the medication was used and the reason for not consulting a doctor was collected. The pattern of drug use over a two-month period preceding the study was noted. Their attitude toward self-medication and source of information for those who practiced self-medication were also recorded. Data were analyzed using EPI Info version 6 and Microsoft Excel and the results were presented using absolute figures and percentages. Analysis was done by using the Chi-square test of significance, to identify the associations among variables.

**Ethical issues:** To obtain the consent of students prior to data collection, a detailed explanation on the aim and objectives of the study was given; and confidentiality was ensured.

### Operational definition

- Self-medication is the selection and use of medicines by individuals to treat self-recognized illnesses or symptoms.

## RESULTS

Four hundred and fourteen students were covered during the study period and 213 (51.4%) of them had faced health-related problems within the last two months prior to the study. Age distribution of those who had episodes of illness in the specified period is shown in Table 1. One hundred and twenty one (56.8%) of 213 were aged between 18 and 20 years, 84 (39.4%) students were aged between 20 and 24 years, and the rest were < 18 years and > 24 years of age, one (0.5%) and seven (3.3%), respectively. One hundred and seventy five (82%) were males and eighty-two (18%) were females. The respective number of students from each year of study is also given in Table 1. Seventy-five (35.2%) were first year students (included first year Medicine, Pharmacy, and Health Science Students), 67 (31.4%) were second year students, 46 (21.6%) were third year students, and 17 (8%) and eight (3.8%) were fourth and fifth year students, respectively. Among the self-medicators 54 (25.4%) were from the School of Medicine, 42 (19.7%) were from the School of Pharmacy, and the remaining 28 (13.1%) and 89 (41.8%) were from the School of Community Health and Health Science, respectively.

**Table 1:** Demographic characteristics of students who reported illness in the last two months in GCMHS, in 2007; N = 213

Variable	Frequency (Percentage)
Sex	
Male (n = 174)	64 (36.6)
Female (n = 39)	18 (46.2)
Age	
17 – 24(n = 122)	45 (37)
21 – 24 (n = 84)	32 (38.1)
> 24 (n = 7)	5 (71.4)
Year	
I year (n = 75)	23 (30.7)
II year (n = 67)	24 (35.8)
III year (n = 46)	14 (30.4)
VI year (n = 17)	14 (82.3)
V year (n = 8)	7 (87.5)
Schools	
Medicine (n = 54)	23 (42.6)
Pharmacy (n = 42)	19 (45.2)
Community health (n = 28)	8 (28.6)
Health sciences (n = 89)	32 (36)

Fever and headache were the most frequently reported causes of morbidity; respiratory and gastrointestinal tract diseases were the second and third most common causes of morbidity, with a frequency of 55 (24.8%), 51 (23.9%), and 28 (13.2%), respectively. Other episodes of illness included diarrhea 19 (8.9%), malaria 13 (6.1%), pneumonia 13 (6.1%), constipation 12 (5.6%), and eye disease 8 (3.8%) [Table 2].

**Table 2:** Frequency of reported symptoms / disease

Type of symptoms /diseases	Frequency (N = 213)	Percent
Fever and Headache	55	25.8
Cough and Common cold	51	23.9
Gastric pain	28	13.2
Diarrhea	19	8.9
Fever and Chills	13	6.1
Cough and chest pain	13	6.1
Constipation	12	5.6
Eye disease	8	3.8
Others*	14	6.5

* Stress, fatigue, loss of appetite, etc.

Eighty-two of the 213 students (38.5%) had practiced self-medication during the two months period preceding the study. As shown in Table 3 among 82 students who practiced self-medication, 59 (72%) obtained drugs from the pharmacy or drug shop without prescription, 13 (5.9%); from their friends, 3 (3.6%); from drugs left over from prior use, and the remaining 7 (8.5%) from plant (traditional medicines). Majority of the students 92 (43.2%) obtained drugs by visiting the physician and with prescription; and 39 (18.3%) accounted for students who suffered episodes of illness and did not take any action

**Table 3:** Measures taken by students who reported an illness (n = 213)

Measure taken	Drug source	Frequency	Percent
Visiting physician	Pharmacy or drug shop with prescription	92	43.2
Self-medication	Pharmacy or drug shop without prescription 59 (72%)	82	38.5
	From friends 13 (15.9%)		
	Drugs left over from prior use 3 (3.6%)		
	Others 7 (8.5%)*		
No action taken	-	39	18.3

* From Relatives, Kiosk, etc., N = 82 for drug source under self-medication

Drugs or drug groups commonly used for self-medication among 82 students is shown in Table 4. The most common drug used in self-care was Paracetamol, that is, 38 (46.3%) of 82 respondents used Paracetamol for self-medication in the preceding two months. Others were analgesics constituting 20 (24.4%), followed by antacids 10 (12.2%), anti-helminthes 9 (10.9%), antibiotics 4 (4.8%), and anti-malarials 3 (3.7%).

**Table 4:** Drugs or drug groups used by the students for self-medication, (n = 82)

Drugs/ drug groups	Frequency	Percent
Paracetamol	38	46.3
NSAIDs	20	24.4
Antacids	10	12.2
Anthelmintics	9	10.9
Antibiotics	4	4.8
Anti-malarials	3	3.7

Among the reasons given for self-medication, 29 (35.4%) respondents felt that they had previous experience of treating a similar illness. Twenty-five (30.5%) respondents felt that the illness was mild and did not require the service of a physician. Eight respondents (9.8%) reported that cost-effectiveness was their major reason to practice self-medication, and 13 (15.8%) stated emergency use [Table 5].

**Table 5:** Factors for self-medication (n = 82)

Reason	Frequency	Percent
Prior experience	29	35.4
Non-seriousness illness	25	30.5
Emergency use	13	15.8
Cost-effectiveness	8	9.8
Others	7	8.5

Information sources to practice self-medication were also analyzed and are shown in Table 6. Respondents who practiced self-medication because of advice given by the physician / nurse, but without prescription covered 13.4% of those who practiced self-medication, and the percentage of those who practiced self-medication because of advice from a pharmacist was 25.6%. Respondents who practiced self-medication within the last two months prior to study because of advice from their friends constituted 19.5% (16 students); and a majority of self-medicators who reported that they did it following information obtained from reading material, traditional healers, and others, constituted 30.5, 3.7, and 7.3%, respectively.

**Table 6:** Information source for those who practiced self-medication (n = 82)

Information source	Frequency	Percent
Reading material	25	30.5
Advice from pharmacist	21	25.6
Advice from friend	16	19.5
Advice from physician / nurse, but without prescription	11	13.4
Advice from traditional healers	3	3.7
Others	6	7.3

Data regarding attitude toward self-medication was collected from 414 students, including those who did not face any health-related problem within two months prior to study [Table 7]; 270 students (55.5%) agreed on the practice of self-medication. On the other hand, 172 (41.5%) students disagreed with this practice.

**Table 7:** Attitude of GCMHS students toward selfmedication practice (n = 414)

Attitude	Frequency	Percent
Agree	270	55.6
Disagree	172	41.5
Others (no comment)	12	2.9

There was no significant difference between the self-medication practices of medical and non-medical students (*P* = 0.57), males and females (*P* = 0.36) or in the school (*P* = 0.46). There was, however, an association between the year of study and self-medication practice (*P* < 0.05).

## DISCUSSION

Self-medication refers to using drugs that have not been prescribed, recommended or controlled by a licensed healthcare specialist.[8] In developing countries people are not only using non-prescription drugs but also prescription drugs, as self-medication products, without supervision.[2] From a previous study in North West Ethiopia, the most common reported factors for self-medication were low severity of symptoms and financial inaccessibility.[6]

Fever and headache were the most commonly reported symptoms in the two-month period prior to the study that led to self-medication, followed by cough and common cold. However, the most prevalent symptoms reported in the previous study in North West Ethiopia were cough and cold followed by fever and headache.[6] As compared with the study result in North West Ethiopia, a higher percent for fever and headache were reported in the present study and an equal percent for cough and cold report (23.9% from both studies).

Of the respondents, 38.5% had taken some form of self-medication during the specified period. In the previous study the prevalence of self-medication varied from 15 to 50% (in Southern Ethiopia and Addis Ababa, respectively).[4,5] The percentage of persons who did not take any action against their illness was relatively lower in this study (18.3%) than in the previous one (27.9%). This difference clearly shows the degree to which people perceive their health-related problems and knowledge about where to go to get relief. Seventy-two percent of the individuals who practiced self-medication reported that they obtained drugs from a pharmacy or drug shops. This indicated that most of the self-medicated persons (72%) had obtained drug-related information (at least when to take, and what should never be taken with the drug) from the dispensers.

Paracetamol and NSAIDs were the most commonly used class of drugs. Antimicrobials were not commonly used for self-medication and were obtained mostly by prescription, and it was relatively low as compared to the prevalence of antibiotics used in Nepal.[9]

Prior experience and non-seriousness of the illness were the two major reasons of self-medication in this study. The low severity of symptoms of illness is frequently reported in literature and different surveys.[6,10] What makes this study different is that the majority of respondents who practiced self-medication reported that they practiced self-medication because of their prior experience.

The major information source for most of those who practiced self-medication was reading material. The result of the present study supported the impact of medical education and knowledge on self-medication practice.

Among the total respondents, 55.5% agreed on self-medication practice and 44.5% disagreed on the practice. The report from the present study, with a mix of medical, pharmacy, and health science students, is relatively low as compared to the report from the medical students of Bahrain, wherein, the majority (76.9%) of the respondents had a positive attitude favoring self-medication.[11]

## CONCLUSIONS

Students in GCMHS, 38.5%, practiced self-medication. Paracetamol and NSAIDs were the drugs most commonly used. Prescription drugs such as antibiotics were involved in self-medication practice. Prior experience and non-seriousness of the illness were the most common reasons for self-medication. Although the self-medication practice is inevitable; drug authorities and health professionals need to educate students about the pros and cons of self medication.
